# Individualized treatment rule for early steroid use in hospitalized patients with community acquired pneumonia: a cohort study

**DOI:** 10.1186/s41479-025-00182-y

**Published:** 2025-11-25

**Authors:** Yewande E. Odeyemi, Allison M. LeMahieu, Erin F. Barreto, Hemang Yadav, Ognjen Gajic, Phillip Schulte

**Affiliations:** 1 Division of Pulmonary and Critical Care Medicine, Mayo Clinic, 200 First Street SW, Rochester, MN 55905 USA; 2 Division of Clinical Trials and Biostatistics, Mayo Clinic, Rochester, MN 55905 USA; 3 Department of Pharmacy, Mayo Clinic, Rochester, MN 55905 USA

**Keywords:** Community-acquired pneumonia, Individualized treatment rule, Corticosteroid, Clinical outcomes

## Abstract

**Background:**

Current evidence on an optimal patient selection strategy for adjunctive steroids to curb excessive inflammation in community acquired pneumonia (CAP) is limited. An individualized treatment rule (ITR) customizes treatment recommendations based on individual patient characteristics. The objective of this study was to develop an ITR for early steroid use in hospitalized patients with CAP.

**Methods:**

Using a single center cohort of hospitalized patients with CAP from 2009 to 2019, we developed a single decision ITR to initiate or not initiate steroids early (within 24 h) after admission. The primary outcome of interest was hospital-free days measured at 28 days. Regression-based learning with LASSO selected a model estimating expected outcomes of potential intervention with steroids individualized to predictors. The optimal ITR was compared to other treatment rules including as observed in the data.

**Results:**

A total of 4379 patients were included in this cohort with 1412 (32%) patients receiving steroids within 24 h of hospital admission. Compared to observed practice, an optimal ITR was associated with increased hospital-free days (mean rate [95% confidence interval (CI)]: 21.74 [21.52, 21.95] versus 22.31 [22.11, 22.51]). The optimal ITR for the secondary outcomes including ventilator-free days, mortality and need for advanced respiratory failure support and/or mortality revealed better estimates compared to what was observed in the data. However, there was a lack of consistent performance when applying the advanced respiratory failure and/or mortality (a secondary outcome) ITR to other outcomes (inconsistency of results across models).

**Conclusion:**

An ITR for early steroid use in hospitalized patients with CAP did not consistently improve clinical outcomes.

**Supplementary Information:**

The online version contains supplementary material available at 10.1186/s41479-025-00182-y.

## Introduction

Community acquired pneumonia (CAP) is a common cause of hospitalization with a significant risk of clinical deterioration and need for advanced support in the intensive care unit (ICU) [1–3]. As inflammation is a key feature of pneumonia and increased inflammatory mediators have been linked to adverse clinical outcomes despite antibiotic use and supportive care, several studies have examined the role of adjunct corticosteroids to curb excessive inflammation [4, 5]. Although recent studies suggest a benefit of corticosteroid in severe CAP, the definition of severity was variable across randomized clinical trials. Moreover, updated guideline recommendations on the use of corticosteroids in CAP are different with one recommending its use in severe CAP and another suggesting a restriction to severe CAP with shock only based on the quality of the evidence available [6–12]. Thus, the use of corticosteroids remains undefined with a lack of optimal patient selection strategies. This has resulted in variability in corticosteroid use in clinical practice beyond common indications like shock or underlying diagnosis of chronic obstructive pulmonary disease (COPD). Despite the increasing attempts at identifying clinical sub phenotypes in related conditions including sepsis and acute respiratory distress syndrome (ARDS) [13–15], strategies focused on optimal patient selection for early corticosteroid use in CAP are still limited [16, 17]. An optimal strategy to identify steroid-responsive phenotypes would offer an opportunity for predictive enrichment into corticosteroid trials in pneumonia, thereby increasing the likelihood of detecting a therapeutic benefit. The use of an effective statistical method for evaluating treatments with heterogeneous effects among patients is therefore important. An individualized treatment rule or regimen (ITR) is a decision rule that informs treatment according to specific patient characteristics, thereby maximizing the overall benefits from a recommended therapy [18, 19]. Estimating ITRs using large scale longitudinal data in a real-world setting provides an opportunity to characterize treatment pathways and inform future treatment recommendations. The objective of this study was to develop an ITR for early steroid use in hospitalized patients with CAP, with treatment recommendation tailored to individual patient characteristics.

## Materials and methods

### Source of data and participants

A single-center retrospective cohort of hospitalized adult (≥ 18 year) patients with CAP from January 2009 to December 2019 was used to develop a single decision ITR to initiate or not initiate corticosteroids early (within 24 h) after admission. Patients were categorized into 2 groups depending on receipt and timing of corticosteroid therapy: Steroid within 24 h (early steroid) and No steroid within 24 h groups (no-early steroid).

The Mayo Clinic Institutional Review Board (IRB) approved this study prior to its initiation (IRB number: 17-011140, modification approval date: June 2021, Title: Concordant versus discordant corticosteroid use with markers of inflammation in critically ill patients with pneumonia and ARDS). Informed consent was waived, and procedures were followed in accordance with the ethical standards of the Mayo Clinic IRB and with the Helsinki Declaration of 1975.

CAP was identified by ICD 9 (481–486) & 10 (J13,15,18) codes and review of clinical notes from the day of admission. Exclusion criteria were lack of research authorization, prior hospitalization within 15 days of admission, aspiration pneumonia, hospital/ventilator acquired pneumonia (diagnosis of pneumonia >48 h of admission or intubation), interstitial lung disease, leukopenia, neutropenia, acquired immunodeficiency syndrome (AIDS) and human immunodeficiency virus (HIV) infection similar to other studies [20].

### Outcome

The primary outcome of interest was hospital-free days calculated as days alive spent outside of the hospital, within 28 days of admission. Hospital-free days was set as 0 for patients who died during the stay or had a length of stay of ≥ 28 days.

Secondary outcomes included:

- Ventilator-free days, calculated as days alive spent not receiving mechanical ventilation (noninvasive and/or invasive) within 28 days of admission. Ventilator free days was set at 0 for patients who died during the stay or had mechanical ventilation duration of ≥ 28 days.
- Intensive care unit (ICU)-free days, calculated as days alive spent outside of the ICU, within 28 days of admission. ICU-free days was set at 0 for patients who died during the stay or had an ICU length of stay of ≥ 28 days.
- Oxygen-free days, calculated as days alive spent not receiving any oxygen therapy within 28 days of admission. Oxygen-free days was set at 0 for patients who died during the stay or had oxygen duration of ≥ 28 days.
- In-hospital mortality.
- Advanced respiratory support and/or mortality.

### Predictors

Patient characteristics may be informative as tailoring variables for the development of the ITR. Potential tailoring variables included age, sex, height, weight, blood pressure, heart rate, respiratory rate, temperature, medical comorbidities (congestive heart failure, asthma, liver disease, neoplastic disease, and renal disease), laboratory data (white blood cell count (WBC) with differentials-neutrophils, eosinophils, lymphocytes; bicarbonate, sodium, BUN; blood gases), clinical scores (pneumonia severity index, SOFA, APACHE III). These predictor variables were applied as follows:

- Early steroid group: data within 6 h of admission and prior to steroid administration.
- No early-steroid group: data within 6 h of admission.

For those predictors measured repeatedly or longitudinally during the first six hours, the first observation was used. Race was grouped as White or other/unknown for analysis. Models adjust for secular trends in time using date of admission as a covariate; date was not allowed to be a tailoring variable since the ITR needs to be generalizable to future data.

### Statistical analysis

Patient demographics, physiological measures, clinical and laboratory measures were summarized using median [interquartile range (IQR)] for continuous variables and frequency (percentage) for categorical variables.

An optimal ITR is that which, if followed, maximizes the primary outcome of hospital-free days on average in the population. In brief, we fit a linear regression model using predictors described previously. Additionally, the model included an indicator for treatment with steroids and includes interactions between steroids and predictors. Because we consider a large number of predictors, a parsimonious model was selected using the Least Absolute Shrinkage and Selection Operator (LASSO) method. Interaction of predictors and steroid group in the model are tailoring variables of the estimated treatment rule. That is, interaction between a predictor and steroid group suggest that the predictor can be used to optimally select steroid group leading to better outcomes when applied to future patients. ITRs are evaluated as the expected outcome if a population were to adhere to the ITR recommendations. We calculated the value of the estimated optimal ITR – the expected hospital-free days, on average– and compared to other treatment rules including (i) physician preference as observed in our data “ Observed practice”, (ii) no early steroid use (“Hypothetical: no steroids”), (iii) always early steroid use (“Hypothetical: all treated with steroids”), We also compare to two regimes reported in a previous study [21]. In brief, the prior study using data from randomized controlled trials estimated that an optimal steroid regimen would give patients steroids based on CRP alone (CRP >204.1 mg/L) or based on a combination of CRP and glucose (glucose >7 mmol/L and CRP >61.8 mg/L, or glucose ≤ 7 mmol/L and CRP >341.2 mg/L). Additionally we utilized the optimal ITR for the outcome of advanced respiratory failure and/or mortality to predict hospital-free days (primary outcome) and other secondary outcomes to test the consistency of models across outcomes. Wilcoxon rank sum tests were used to assess the statistical difference between observed and optimal ITRs.

A similar approach was used for ventilation-free days, ICU-free days, and oxygen-free days. In-hospital mortality and need for advanced respiratory support and/or mortality was evaluated using a similar approach, but with a logistic regression model. Sensitivity analyses assessed primary and secondary outcomes excluding (1) patients with COPD and (2) patients with COPD or recipients of late steroids (more than 24 h into hospitalization). Missing data for predictor variables were multiply imputed 50 times using multivariate imputation by chained equations. There was no missing data for primary or secondary outcomes; missing data of predictors is summarized in Supplementary Table 1. The estimated optimal ITR is compared to observed practice outcomes using a Wilcoxon signed rank test; *p* < 0.05 is considered statistically significant. Data management and analysis were performed in SAS version 9.4 (SAS Institute Inc, Cary, North Carolina) and R version 4.1.2 (RStudio Team 2021, Boston, Massachusetts). Additional detailed statistical methods can be found in the Supplemental Methods.

## Results

**Fig. 1:**
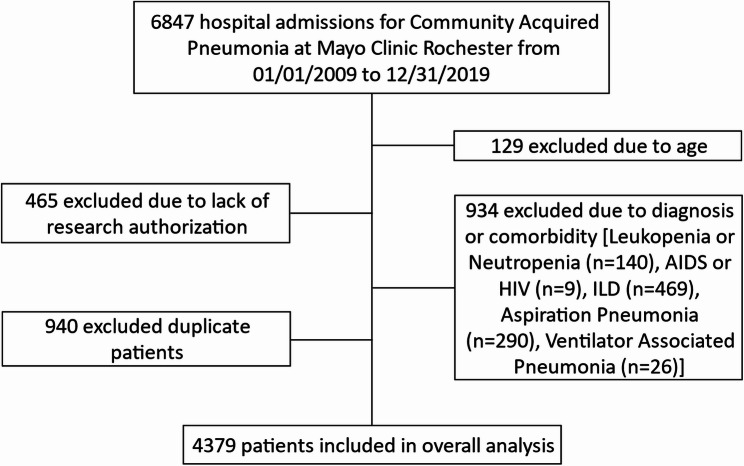
Study Flow Diagram

A total of 4379 patients were included in this cohort (Fig. 1), 1,412 (32%) patients received steroids within 24 h of hospital admission (steroid group) and 2,967 (79%) either received steroids after 24 h or received no steroids during hospitalization (no early steroid group). The timing of steroids relative to admission is shown in Supplementary Fig. 1. Demographics and clinical characteristics by group are shown in Table 1. Patients in the steroid group were significantly younger compared to the no-steroid group (median [IQR] = 71.5 [59.9, 80.4] and 74.8 [61.8, 84.6]) and more likely to have an underlying diagnosis of COPD (58% versus 25%) or Asthma (23% versus 13%). A higher proportion of patients received vasopressors within the first 6 h of admission in the steroid group compared to the no-steroid group (11% versus 6%, p value < 0.001). The extent of missing data prior to imputation is described in Supplemental Table 1.

**Table 1:** Demographics and clinical characteristics for all patient RST hospitalizations, *N* = 4379

	Early steroids (*n* = 1412)	No early steroids (*n* = 2967)	*p*-value
Gender, n (%)			0.57
Female	641 (45)	1374 (46)	
Male	771 (55)	1593 (54)	
Age, median (IQR)	71.5 (59.9, 80.4)	74.8 (61.8, 84.6)	< 0.001
Race, n (%)			0.14
American Indian/Alaskan Native	4 (0)	15 (1)	
Asian	23 (2)	28 (1)	
Black or African American	26 (2)	41 (1)	
Other	33 (2)	50 (2)	
Unknown	8 (1)	20 (1)	
White	1318 (93)	2811 (95)	
Comorbidities, n (%)			
Altered mental status	139 (10)	395 (13)	0.001
Asthma	320 (23)	344 (12)	< 0.001
COPD	821 (58)	744 (25)	< 0.001
Congestive heart failure	433 (31)	901 (30)	0.84
Liver disease	40 (3)	98 (3)	0.41
Neoplastic disease	508 (36)	1141 (39)	0.11
Renal disease	397 (28)	897 (30)	0.15
First physical-examination, median (IQR)			
Weight (kg), *N* = 3997	78.2 (63.5, 95.7)	78.9 (65.2, 95.6)	0.18
Diastolic BP (mmHg), *N* = 4286	70.0 (60.0, 82.0)	69.0 (59.0, 81.0)	0.26
Systolic BP (mmHg), *N* = 4285	127.0 (110.0, 144.0)	127.0 (112.0, 145.0)	0.32
Pulse (bpm), *N* = 4260	94.0 (81.0, 108.0)	89.0 (76.0, 103.0)	< 0.001
Respiratory rate (cpm), *N* = 2760	22.0 (18.0, 27.0)	20.0 (18.0, 24.0)	< 0.001
Temperature (°C), *N* = 4230	36.8 (36.6, 37.2)	36.8 (36.6, 37.2)	0.73
Lab and radiologic findings, median (IQR)			
Blood urea nitrogen (mg/dl), *N* = 4087	20.0 (14.0, 29.0)	21.0 (14.0, 31.0)	0.08
Bicarbonate (mmol/l), *N* = 3920	26.0 (23.0, 29.0)	25.0 (22.0, 28.0)	< 0.001
Glucose (mg/dl), *N* = 4125	131.0 (110.0, 167.0)	128.0 (108.0, 163.0)	0.18
Sodium (mmol/l), *N* = 4148	138.0 (134.0, 140.0)	137.0 (134.0, 140.0)	0.008
Hematocrit (%), *N* = 4135	38.4 (34.2, 42.0)	37.0 (33.0, 40.9)	< 0.001
WBC (x 10^9^/l), *N* = 4128	12.0 (8.6, 16.0)	11.8 (8.6, 15.8)	0.26
Neutrophils (x 10^9^/l), *N* = 3631	9.8 (6.8, 13.5)	9.4 (6.5, 13.1)	0.08
Eosinophils (x 10^9^/l), *N* = 2552	0.1 (0, 0.2)	0.1 (0, 0.2)	0.29
N/L ratio, *N* = 3612	11.0 (5.9, 19.5)	9.1 (5.3, 15.4)	< 0.001
CRP (mg/l), *N* = 339	77.7 (35.3, 145.3)	94.8 (33.3, 187.0)	0.33
Partial pressure of arterial oxygen (mmHg), *N* = 1555	69.0 (48.0, 93.0)	67.0 (43.0, 91.3)	0.31
Arterial pH, *N* = 1711	7.37 (7.29, 7.42)	7.38 (7.33, 7.43)	< 0.001
Pleural effusion, n (%), *N* = 581	86 (49)	217 (53)	0.39
Initial clinical scores, median (IQR)			
Pneumonia severity index	115.0 (87.0, 145.0)	114.0 (90.0, 142.0)	0.76
CURB-65	3.0 (2.0, 3.0)	3.0 (2.0, 3.0)	0.46
SOFA, *N* = 1812	4.0 (2.5, 7.0)	4.0 (2.0, 6.5)	0.047
APACHE III, *N* = 1812	50.0 (39.0, 66.0)	50.0 (39.0, 63.0)	0.54
ICU admit at six hours, n (%)	593 (42)	844 (28)	< 0.001
Vasopressors within six hours of admission, n (%)	153 (11)	173 (6)	< 0.001

Data are summarized using median [interquartile range (IQR)] for continuous variables and frequency (percentage) for categorical variables

*BP* blood pressure, *CRP * C-reactive protein,* WBC* white blood cell, *N/L rato* neutrophil/lymphocyte ratio

### Primary outcome

Overall mortality was 3.5% and 14% had advanced respiratory support or mortality (Supplementary Table 2). Table 2 outlines the ITR model results for the primary and secondary outcomes compared to the value of other treatment rules including (i) “Observed practice”, (ii) “Hypothetical: no steroids” (iii) “Hypothetical: all treated with steroids”, and (iv) regimes reported in previous studies [21]. We expect that under the optimal ITR, mean hospital free days would be 22.31 (95%confidence interval [CI]: 22.11, 22.51) [Table 2]. In contrast, under current medical practice (observed practice)) we observed 21.74 (95%CI: 21.52, 21.95) hospital-free days. “hypothetical: no steroid” and “hypothetical: all treated with steroid” rules also performed poorly relative to the optimal rule (21.50 (95%CI: 21.28, 21.71) and 22.00 (95%CI: 21.80, 22.21)). Furthermore, in comparison to a previously reported regimen [21], our estimated ITR demonstrated superior performance. However, there was a lack of consistency of superior performance utilizing the specific optimal ITR for the outcome of advanced respiratory failure and/or mortality (a secondary outcome) to predict hospital-free days (inconsistency of results across models). When comparing observed and optimal ITRs, mean hospital-free days were statistically significant different (*p* < 0.001). Full ITR model results for the outcome of hospital-free days are shown in Supplemental Table 5.

**Table 2:** Individualized treatment rule model results

	Observed practice	Hypothetical: no steroids	Hypothetical: all treated with steroids	Optimal treatment	CRP regimen^a^	CRP and glucose regimen^b^	Advanced respiratory support or mortality model
Hospital-free days	21.74 (21.52, 21.95)	21.50 (21.28, 21.71)	22.00 (21.8, 22.21)	22.31 (22.11, 22.51)	21.65 (21.43, 21.88)	21.68 (21.47, 21.89)	21.95 (21.74, 22.16)
Ventilation-free days	26.61 (26.46, 26.76)	26.66 (26.52, 26.80)	26.60 (26.43, 26.76)	27.08 (26.93, 27.22)	26.67 (26.52, 26.83)	26.66 (26.51, 26.81)	26.56 (26.41, 26.72)
ICU-free days	26.52 (26.39, 26.66)	26.50 (26.37, 26.64)	26.51 (26.37, 26.66)	26.84 (26.71, 26.98)	26.52 (26.38, 26.67)	26.53 (26.39, 26.67)	26.57 (26.43, 26.71)
Oxygen-free days	24.60 (24.40, 24.79)	24.42 (24.23, 24.61)	24.91 (24.71, 25.10)	25.19 (25.00, 25.37)	24.56 (24.36, 24.75)	24.60 (24.41, 24.79)	24.78 (24.59, 24.98)
Mortality (%)	3.73 (3.23, 4.24)	3.49 (3.01, 3.97)	3.72 (3.21, 4.22)	2.12 (1.81, 2.42)	3.41 (2.88, 3.94)	3.38 (2.88, 3.87)	3.94 (3.42, 4.47)
Advanced respiratory support or mortality (%)	12.47 (11.28, 13.67)	13.76 (12.50, 15.01)	10.42 (9.28, 11.57)	8.62 (7.68, 9.56)	13.46 (12.14, 14.78)	13.15 (11.88, 14.42)	8.62 (7.68, 9.56)

Estimates summarized using mean [Confidence interval (CI)]. ^a^ Final model 1 based on CRP alone (CRP >204.1 mg/L) by ITR regimen published by Smit et al. [21]. ^b^ Final model 3 based on combination of CRP and glucose (glucose >7 mmol/L and CRP >61.8 mg/L, or glucose ≤ 7 mmol/L and CRP >341.2 mg/L) by ITR regimen published by Smit et al. [21]. P-values are reported comparing Optimal Treatment ITR to Observed Practice: Hospital-free days *p* < 0.001; Ventilation-free days *p* < 0.001; ICU-free days *p* < 0.001; Oxygen-free days *p* < 0.001; Mortality (%) *p* < 0.001; Advanced respiratory support or mortality (%) *p* < 0.001

###  Secondary outcomes

Under the optimal ITR, we expect the mean ventilation-free days would be 27.08 (95% CI: 26.93, 27.22). [Table 2]. In contrast, mean ventilation-free days in our observed data (observed practice) was 26.61 (95% CI: 26.46, 26.76), similar to the “hypothetical: no steroid” and” hypothetical: all treated with steroid” rules (26.66 (95% CI: 26.52, 26.80), and 26.60 (95% CI: 26.43, 26.76) respectively).

We expect the mean ICU-free days under the optimal ITR would be 26.84 (95% CI: 26.71, 26.98. However, the mean ICU-free days in our observed data was 26.52 (95% CI: 26.39, 26.66), similar to the hypothetical: no steroid” and” hypothetical: all treated with steroid” ((26.50 (95% CI: 26.37, 26.64) and 26.51 (95% CI: 26.37, 26.64)).

With the optimal ITR, we expect the mean oxygen-free days would be 25.19 (95% CI: 25.00, 25.37). The observed, mean oxygen-free days in our data was 24.60 (95% CI: 24.40, 24.79). If no patients received steroids “hypothetical: no steroid”, estimated mean oxygen-free days would be 24.42 (95% CI: 24.23, 24.61), while if all patients received steroids “hypothetical: all treated with steroid”, the estimated mean would be 24.91 oxygen-free days (95% CI: 24.71, 25.10).

Under the optimal ITR, the expected mean mortality rate would be 2.12% (95% CI: 1.81, 2.42) while the observed mean mortality rate was 3.73% (95% CI: 3.23, 4.24).

Using the optimal ITR, the expected mean rate of advanced respiratory support or mortality would be 8.62% (95% CI: 7.68, 9.56) compared to the observed mean rate of 12.47% (95% CI: 11.28, 13.67).

**Fig. 2:**
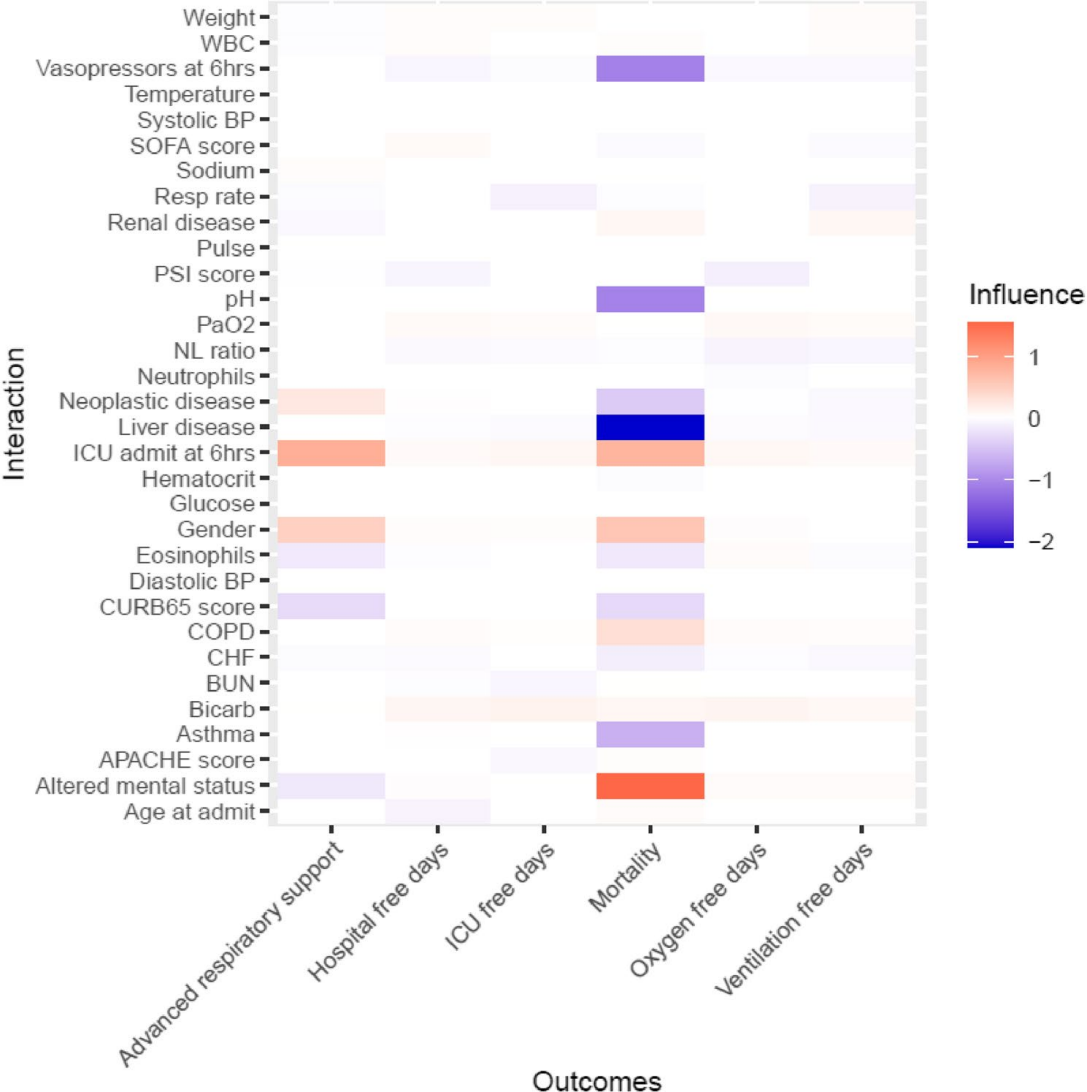
Heat map of model interactions. Heat map of model interaction between steroid treatment and tailoring variables. WBC = white blood cell. BP = blood pressure. Resp rate = respiratory rate. Pulse = pulse rate. NL ratio = neutrophil/lymphocyte ratio. CHF – congestive heart failure. BUN = blood, urea, nitrogen. Bicarb = bicarbonate. >0 = Positive influence (decreased need for advanced respiratory support and/or mortality risk and increased-free days). < 0 = Negative influence (increased need for advanced respiratory support and/or mortality risk and decreased free-days

In comparison to a previously reported regimen [21], our estimated optimal ITR demonstrated superior performance for all secondary outcomes. However, there was a lack of consistent performance when applying the advanced respiratory failure and/or mortality (a secondary outcome) ITR to other outcomes (inconsistency of results across models). This inconsistency is further demonstrated in a heatmap which illustrates which variables were selected in different outcome ITRs and the saturation of color indicates importance or strength of the predictor (Fig. 2). The differences in mean value for all secondary outcomes were statistically significant when comparing observed and optimal ITRs (all *p* < 0.001).

Results for sensitivity analyses excluding patients with COPD and excluding COPD and those with steroids started > 24 h after admission are shown in Supplemental Tables 3 and Supplemental Table 4, respectively. Overall, the optimal treatment ITR suggested improved outcomes, but with similar inconsistency in results.

## Discussion

In this single-center retrospective cohort of hospitalized patients with CAP, we found that an optimal ITR for early corticosteroid use was not associated with a consistent improvement in clinical outcomes. Although the optimal ITR was significantly associated with improved clinical outcomes (primary and secondary outcomes, including mortality) compared to the observed practice, there was a lack of consistency across models limiting utility and reliability for practice. For example, the prediction of hospital-free days was different between an optimal ITR focused on maximizing the outcome of hospital-free days and an optimal ITR focused on the need for advanced respiratory support and/or mortality. It would be expected that the significant improvement noted with an optimal ITR for advanced respiratory failure and/or mortality (about 30% decrease) would translate to an improvement in hospital-free days. However, this concordance was not observed in our study. Overall, these data do not currently support the use of estimated ITRs to help guide corticosteroid use in CAP.

ITR in critical care has been described in few studies including pertaining to fluid strategy in sepsis, corticosteroid in septic shock and corticosteroids in CAP [22, 23]. Recently, an ITR model was developed and validated to predict individualized treatment effect of corticosteroids on 30-day mortality in hospitalized patients with CAP using individual patient data from six randomized clinical trials comparing corticosteroid with placebo (n −1,869). In this study, the models identified high baseline C-reactive protein (CRP) and glucose levels as major predictors of a good response to corticosteroids. In a subsequent study including two additional randomized control trials for external validation, CRP was again identified as a major effect modifier with mortality benefit of corticosteroid observed with high CRP levels (>204 mg/L) [24]. The external evaluation of these models on our data revealed inferior results compared to our early steroid ITR model in this study. Noteworthy are important distinctions between our models and their models. First, the focus on early (within 24 h of admission) steroid use in our model compared to steroid use in 12–36 h in the randomized clinical trials used to develop and validate the models by Smit el. In our study, an ITR to initiate corticosteroid in the first 24 h was intentionally prespecified to both reflect the impact of early treatment strategies on outcome and the practical use of the ITR as a predictive enrichment strategy in randomized clinical trials where early interventions are targeted. Secondly, our ITR was developed using retrospective data compared to the individual patient data from randomized controlled trials used by Smit al. Data from randomized clinical trials are typically used to estimate average treatment effects, however, they are insufficient to facilitate individual treatment decision in practice. The goal of an ITR is to select a treatment strategy that would optimize a patient’s outcome based on patient-specific characteristics and decrease the chances of side effects. This tailoring of treatment facilitates a precision medicine approach. ITR based on randomized clinical trial are often limited by the strict inclusion and exclusion criteria therefore studies have evaluated the use of real-world data in estimating ITRs [18, 19, 23, 25, 26]. However, a major limitation with the use of electronic health record data is the lack of random assignments and inability to infer causality due to its inherent selection and unmeasured confounding biases that could result in either overestimation or underestimation of an effect. Thus, the utilization of a valid statistical method is critical in constructing an optimal ITR from observational data to potentially deal with confounding variables and heterogeneity. Although a variety of statistical methods have been developed to draw inferences from observational data, the use of a regression-based learning with LASSO as described in this study offers a less biased methodology to estimate an ITR. Thirdly, due to the retrospective nature of the study, data on CRP in the first 6 h was mostly unavailable therefore CRP was excluded as a predictor variable and did not inform our early steroid ITR compared to the study by Smit et al. Despite these differences, our optimal ITR models had a superior performance over the reported regimens by Smit et al. [21] but its lack of consistency across clinical outcomes limits its clinical use.

Since this was a single center study, findings presented in this paper are limited to the characteristics as seen in a large academic referral center. Additional prospective validation or validation with other medical centers would be appropriate before implementing a model in practice. This retrospective study also lacked information on review of chest x-ray findings to confirm the diagnosis of pneumonia and information on other potential causes of immunosuppression including the use of disease- modifying antirheumatic drugs. An additional limitation is the unavailability or high missingness of clinical variables in the first 6 h that may inform corticosteroid use in pneumonia including CRP as demonstrated in prior studies [16, 17]. Other unmeasured parameters at the time of admission may have an important role in predicting steroid responsiveness.

## Conclusion

In this retrospective cohort study, an ITR for early corticosteroid use in hospitalized patients with CAP did not consistently improve prediction of clinical outcomes.

## Supplementary Information

Supplementary Material 1

Supplementary Material 2

## Data Availability

The datasets used and/or analyzed during the current study are available from the corresponding author on reasonable request.

## Abbreviations

AIDS: Acquired immunodeficiency syndrome
APACHE: Acute physiology and chronic health evaluation
BUN: Blood urea nitrogen
C.I: Confidence interval
CAP: Community-acquired pneumonia
COPD: Chronic obstructive pulmonary disease
CRP: C-reactive protein
HIV: Human immunodeficiency
HFD: Hospital-free days
ITR: Individualized treatment rule
ICD: International Classification of Diseases
ICU: Intensive care unit
IQR: Interquartile range
OR: Odds ratio
LASSO: Least absolute shrinkage and selection operator
PSI: Pneumonia severity index
SOFA: Sequential organ failure assessment
WBC: White blood cell count

## References

[1] Phua J, Ngerng WJ, Lim TK. The impact of a delay in intensive care unit admission for community-acquired pneumonia. Eur Respir J. 2010;36(4):826–33. 20185424 10.1183/09031936.00154209

[2] Aliberti S, Brambilla AM, Chalmers JD, Cilloniz C, Ramirez J, Bignamini A, et al. Phenotyping community-acquired pneumonia according to the presence of acute respiratory failure and severe sepsis. Respir Res. 2014;15(1):27. 24593040 10.1186/1465-9921-15-27 PMC4015148

[3] Odeyemi YE, Lal A, Barreto EF, LeMahieu AM, Yadav H, Gajic O et al. Early machine learning prediction of hospitalized patients at low risk of respiratory deterioration or mortality in community-acquired pneumonia: derivation and validation of a multivariable model. Biomol Biomed. 2024;24(2):337-45. 10.17305/bb.2023.9754 PMC10950343 37795970

[4] Martinez R, Menendez R, Reyes S, Polverino E, Cilloniz C, Martinez A, et al. Factors associated with inflammatory cytokine patterns in community-acquired pneumonia. Eur Respir J. 2011;37(2):393–9. 20595152 10.1183/09031936.00040710

[5] Burke H, Freeman A, Cellura DC, Stuart BL, Brendish NJ, Poole S, et al. Inflammatory phenotyping predicts clinical outcome in COVID-19. Respir Res. 2020;21(1):245. 32962703 10.1186/s12931-020-01511-z PMC7506817

[6] Pastores SM, Annane D, Rochwerg B. Guidelines for the diagnosis and management of critical illness-Related corticosteroid insufficiency (CIRCI) in critically ill patients (Part II): society of critical care medicine (SCCM) and European society of intensive care medicine (ESICM) 2017. Crit Care Med. 2018;46(1):146–8. 29095205 10.1097/CCM.0000000000002840

[7] Metlay JP, Waterer GW, Long AC, Anzueto A, Brozek J, Crothers K, et al. Diagnosis and treatment of adults with Community-acquired Pneumonia. An official clinical practice guideline of the American thoracic society and infectious diseases society of America. Am J Respir Crit Care Med. 2019;200(7):e45–67. 31573350 10.1164/rccm.201908-1581ST PMC6812437

[8] Chaudhuri D, Nei AM, Rochwerg B, Balk RA, Asehnoune K, Cadena R et al. 2024 focused update: guidelines on use of corticosteroids in Sepsis, acute respiratory distress Syndrome, and Community-Acquired pneumonia. Crit Care Med 9900:10.1097/CCM.0000000000006172 10.1097/CCM.0000000000006172 38240492

[9] Martin-Loeches I, Torres A, Nagavci B, Aliberti S, Antonelli M, Bassetti M, et al. ERS/ESICM/ESCMID/ALAT guidelines for the management of severe community-acquired pneumonia. Intensive Care Med. 2023;49(6):615–32. 37012484 10.1007/s00134-023-07033-8 PMC10069946

[10] Cheema HA, Musheer A, Ejaz A, Paracha AA, Shahid A, Rehman MEU, et al. Efficacy and safety of corticosteroids for the treatment of community-acquired pneumonia: A systematic review and meta-analysis of randomized controlled trials. J Crit Care. 2024;80:154507. 38128217 10.1016/j.jcrc.2023.154507

[11] Xin YS, Tsu Hsien W, Yu-Cheng C, Juien L, Weitao L, Cheryn Yu Wei C, et al. Impact of different corticosteroids on severe community-acquired pneumonia: a systematic review and meta-analysis. BMJ Open Respiratory Res. 2024;11(1):e002141. 10.1136/bmjresp-2023-002141 PMC10806634 38262670

[12] Lu D-E, Chang C-Y, Cheng S-W, Kang E, Lee C-H, Chen K-H. Evidence supports the use of hydrocortisone for patients with community-acquired pneumonia. Crit Care. 2024;28(1):55. 38378580 10.1186/s13054-024-04833-2 PMC10880276

[13] Sinha P, Furfaro D, Cummings MJ, Abrams D, Delucchi K, Maddali MV, et al. Latent class analysis reveals COVID-19-related acute respiratory distress syndrome subgroups with differential responses to corticosteroids. Am J Respir Crit Care Med. 2021;204(11):1274–85. 34543591 10.1164/rccm.202105-1302OC PMC8786071

[14] Li H, Markal A, Balch JA, Loftus TJ, Efron PA, Ozrazgat-Baslanti T, et al. Methods for phenotyping adult patients in sepsis and septic shock: A scoping review. Crit Care Explorations. 2022;4(4):e0672. 10.1097/CCE.0000000000000672 PMC8970078 35372844

[15] Calfee CS, Delucchi K, Parsons PE, Thompson BT, Ware LB, Matthay MA. Subphenotypes in acute respiratory distress syndrome: latent class analysis of data from two randomised controlled trials. Lancet Respiratory Med. 2014;2(8):611–20. 10.1016/S2213-2600(14)70097-9 PMC4154544 24853585

[16] Odeyemi YE, Herasevich S, Chalmers SJ, Barreto EF, Frank RD, Gajic OO, et al. Biomarker-Concordant steroid use in critically ill patients with pneumonia. Mayo Clin Proc Innov Qual Outcomes. 2020;4(6):649–56. 33367210 10.1016/j.mayocpiqo.2020.07.011 PMC7749267

[17] Odeyemi YE, Chalmers SJ, Barreto EF, Jentzer JC, Gajic O, Yadav H. Early, biomarker-guided steroid dosing in COVID-19 pneumonia: a pilot randomized controlled trial. Crit Care. 2022;26(1):9. 34983600 10.1186/s13054-021-03873-2 PMC8724733

[18] Wu P, Zeng D, Wang Y. Matched learning for optimizing individualized treatment strategies using electronic health records. J Am Stat Assoc. 2020;115(529):380–92. 33041401 10.1080/01621459.2018.1549050 PMC7539620

[19] Laber EB, Davidian M. Dynamic treatment regimes, past, present, and future: A conversation with experts. Stat Methods Med Res. 2017;26(4):1605–10. 28482753 10.1177/0962280217708661 PMC5519448

[20] Barlow G, Nathwani D, Davey P. The CURB65 pneumonia severity score outperforms generic sepsis and early warning scores in predicting mortality in community-acquired pneumonia. Thorax. 2007;62(3):253–9. 16928720 10.1136/thx.2006.067371 PMC2117168

[21] Smit JM, Van Der Zee PA, Stoof SCM, Van Genderen ME, Snijders D, Boersma WG et al. Predicting individualized treatment effects of corticosteroids in community-acquired-pneumonia: a data-driven analysis of randomized controlled trials. medRxiv. 2023:2023.10.03.23296132.

[22] Zhang Z, Zheng B, Liu N. Individualized fluid administration for critically ill patients with sepsis with an interpretable dynamic treatment regimen model. Sci Rep. 2020;10(1):17874. 33087760 10.1038/s41598-020-74906-z PMC7578643

[23] Pirracchio R, Hubbard A, Sprung CL, Chevret S, Annane D, Collaborators RRCRSS. Assessment of machine learning to estimate the individual treatment effect of corticosteroids in septic shock. JAMA Netw Open. 2020;3(12):e2029050–e. 33301017 10.1001/jamanetworkopen.2020.29050 PMC7729430

[24] Smit JM, Van Der Zee PA, Stoof SCM, Van Genderen ME, Snijders D, Boersma WG et al. Predicting benefit from adjuvant therapy with corticosteroids in community-acquired pneumonia: a data-driven analysis of randomised trials. The Lancet Respiratory Medicine. 2025;13(3):221-33. 10.1016/S2213-2600(24)00405-3 39892408

[25] Hripcsak G, Ryan PB, Duke JD, Shah NH, Park RW, Huser V, et al. Characterizing treatment pathways at scale using the OHDSI network. Proc Natl Acad Sci U S A. 2016;113(27):7329–36. 27274072 10.1073/pnas.1510502113 PMC4941483

[26] Schulte PJ, Tsiatis AA, Laber EB, Davidian M. Q- and A-learning methods for estimating optimal dynamic treatment regimes. Stat Sci. 2014;29(4):640–61. 25620840 10.1214/13-STS450 PMC4300556

