# Supplementary material for: Individualized treatment rule for early steroid use in hospitalized patients with community acquired pneumonia: a cohort study
Source: Pneumonia (Nathan). 2025 Nov 25;17:29. doi: 10.1186/s41479-025-00182-y (PMC12645675; doi:10.1186/s41479-025-00182-y)
Supplement: Supplementary file 1 — Supplementary Material 1 [file 41479_2025_182_MOESM1_ESM.docx]

**SUPPLEMENTAL METHODS**

Define treatment group for the $i$th subject as $A_{i}=0$ if no steroids group and $A_{i}=1$ if steroids group. Define potential outcomes for the $i$th patient $Y_{i}^{*}(0)$ and $Y_{i}^{*}(1)$ as the outcomes potentially observed if the $i$th patient received no steroids or steroids, respectively. We make assumptions including the stable unit treatment value assumption (SUTVA), the treatment assignment mechanism is independent of the potential outcomes conditional on other observed data (strong ignorability), and all patients in the population have positive probability, given their covariates, or steroids and no steroids (positivity)(1)).

We define a treatment rule $d\left( X \right)$ which takes potential inputs $X$ and maps to an output recommending $A\in\left\{ 0,1 \right\}$. The treatment rule may be a function of $X$, or may be independent of $X$ such as a rule $d\left( X \right)=1$ which outputs the steroid group recommendation for all patients. We also define potential outcomes under a treatment rule, $Y^{*}\left( d \right)$. In the current study, we use a regression-estimator to estimate an optimal treatment rule often called the Q-function: $Q\left( \boldsymbol{x},a \right)= E\left[ Y | \boldsymbol{X}=\boldsymbol{x},A=a \right]=\beta_{0}+\boldsymbol{x}_{\boldsymbol{1}}^{\boldsymbol{T}}\beta_{1}+{a\beta}_{2}+ {a\boldsymbol{x}_{\boldsymbol{2}}^{\boldsymbol{T}}\beta}_{3}$ where $\boldsymbol{x}_{\boldsymbol{1}}$ and $\boldsymbol{x}_{\boldsymbol{2}}$ may be a vector of covariates with $\boldsymbol{x}_{\boldsymbol{1}},\boldsymbol{x}_{\boldsymbol{2}}\in\boldsymbol{x}$. That is, the covariates with a main effect term and those with an interaction with treatment term may be the same or may contain different covariates.

We quantify the value of a treatment rule as the expected outcome if a population were to adhere to the recommendations of the treatment rule. That is, $V\left( d \right)=E[Y^{*}\left( d \right)]$. An optimal treatment rule, which we aim to estimate, maximizes the expected outcome (such as hospital free days): $d^{opt}=argmax_{d} V(d)$. Since larger values of hospital free days are better, we see from the regression equation above that $d^{opt}\left( X \right)=I(\beta_{2}+ {\boldsymbol{x}_{\boldsymbol{2}}^{\boldsymbol{T}}\beta}_{3}>0)$ where $I(.)$ is an indicator function assigning 0 when false and 1 when true. And from this, we see that $\boldsymbol{x}_{\boldsymbol{2}}$ are tailoring variables which tailor the estimated optimal treatment rule to the individual.

After estimating the regression equation (Q-function) described previously and thus estimating the treatment rule, we use a regression-based estimator for the value of the regime. Specifically, we see that $V\left( d \right)=E\left[ Y^{*}\left( d \right) \right]=E\left[ Q\left( \boldsymbol{x},1 \right)I\left( d\left( X \right)=1 \right)+Q\left( \boldsymbol{x},0 \right)I\left( d\left( X \right)=0 \right) \right].$

There are two issues when using a single dataset to estimate an optimal treatment regime and then describe the quality or value of that regime. First, when evaluating the value of a treatment regime, estimating the regime and evaluating value in the same dataset may lead to biased inference on the value of the estimated optimal regime. That is, the regression may be overfitted to the training data yielding an overly optimistic evaluation of performance. Second, the value function described previously is non-regular since the treatment rule is a non-smooth function of the data. This means that both standard asymptotic confidence intervals and non-parametric bootstrap confidence intervals perform poorly to describe the value of the estimated regime. However, we resolve both using a split-sample training and testing data sets. The first issue is clearly resolved when the model is fitted to data that are not used to evaluate. Further, when the estimated treatment rule is applied to the test data, it is fixed and therefore, performance is asymptotically normal.(1) A 70%/30% random split was used for training and testing.

Missing data were present among potential covariates in the Q-function, including among potential tailoring variables. Missing data were multiply imputed, using 50 imputations implemented using multiple imputation with chained equations using predictive mean matching and logistic regression for binary and categorical (generalized logit). We assume that in a practice scenario implementing the optimal treatment rule, data will be fully ascertained.

We sought a parsimonious Q-function using a subset of the covariates most strongly associated with the outcome (hospital free days). In the training data, we use least absolute shrinkage and selection operator (LASSO) to fit the Q-function. In the presence of missing data, we used the Stacked LASSO(2, 3). The one-standard-error rule was applied to be more inclusive of potential tailoring variables.(4) When the estimated treatment rule is applied to the test data to calculate the value of the treatment rule, we use the standard Rubin’s rules for calculating variance and subsequent confidence intervals.(5)

The same approach was used for each outcome separately. In the case of mortality, an optimal rule minimizes the expected outcome proportion, and the correction is easily applied.

1. Anastasios A. Tsiatis MD, Shannon T. Holloway, Eric B Laber. Dynamic Treatment Regimes, Statistical Methods for Precision Medicine. 1st ed. New York: Taylor and Francis Group; 2019 18 December 2019. 618 p.

2. Du J, Boss J, Han P, Beesley LJ, Kleinsasser M, Goutman SA, et al. Variable selection with multiply-imputed datasets: choosing between stacked and grouped methods. J Comput Graph Stat. 2022;31(4):1063-75.

3. Gunn HJ, Hayati Rezvan P, Fernández MI, Comulada WS. How to apply variable selection machine learning algorithms with multiply imputed data: A missing discussion. Psychol Methods. 2023;28(2):452-71.

4. Trevor Hastie RT, Jerome Friedman. The elements of statistical learning: data mining, inference, and prediction. Vol. 2. New York: springer, 2009. New York: Springer Nature; 2009.

5. Shen J, Hubbard RA, Linn KA. Estimation and evaluation of individualized treatment rules following multiple imputation. Stat Med. 2023;42(23):4236-56.
