# Supplementary material for: Individualized treatment rule for early steroid use in hospitalized patients with community acquired pneumonia: a cohort study
Source: Pneumonia (Nathan). 2025 Nov 25;17:29. doi: 10.1186/s41479-025-00182-y (PMC12645675; doi:10.1186/s41479-025-00182-y)

**Supplementary table 1: Summary of missing data**

|  | Observed | Unobserved |
| --- | --- | --- |
| Gender | 4379 | 0 |
| Age | 4379 | 0 |
| Race | 4379 | 0 |
| Comorbidities | 4379 | 0 |
| First physical-examination |  |  |
| Weight | 3997 | 382 |
| Diastolic BP | 4286 | 93 |
| Systolic BP | 4285 | 94 |
| Pulse rate | 4260 | 119 |
| Respiratory rate | 2760 | 1619 |
| Temperature (°C) | 4230 | 149 |
| Lab and radiologic findings |  |  |
| Blood urea nitrogen | 4087 | 292 |
| Bicarbonate | 3920 | 459 |
| Glucose | 4125 | 254 |
| Sodium | 4148 | 231 |
| Hematocrit | 4135 | 244 |
| WBC | 4128 | 251 |
| Neutrophils | 3631 | 748 |
| Eosinophils | 2552 | 1827 |
| N/L ratio | 3612 | 767 |
| CRP | 339 | 4040 |
| Partial pressure of arterial oxygen | 1555 | 2824 |
| Arterial pH | 1711 | 2668 |
| Pleural effusion | 581 | 3798 |
| Initial clinical scores |  |  |
| Pneumonia severity index | 4379 | 0 |
| CURB-65 | 4379 | 0 |
| SOFA | 1812 | 2567 |
| APACHE III | 1812 | 2567 |
| Vasopressors within six hours of admission | 4379 | 0 |

BP = blood pressure. CRP = C-reactive protein. WBC = white blood cell. N/L rato = neutrophil/lymphocyte ratio

**Supplementary table 2: Summary of outcomes**

|  | Overall (n=4379) | Early Steroids (n=1412) | No early steroids (n=2967) | p-value |
| --- | --- | --- | --- | --- |
| Hospital-free days, median (IQR) | 23.88 (20.73, 25.37) | 24.07 (21.03, 25.55) | 23.75 (20.55, 25.32) | 0.71 |
| Ventilation-free days, median (IQR) | 28.00 (28.00, 28.00) | 28.00 (27.48, 28.00) | 28.00 (28.00, 28.00) | 0.001 |
| ICU-free days, median (IQR) | 28.0 (26.67, 28.0) | 27.81 (26.11, 28.00) | 28.00 (26.92, 28.00) | <0.001 |
| Oxygen-free days, median (IQR) | 27.23 (24.49, 27.75) | 27.11 (24.56, 27.75) | 27.32 (24.45, 27.75) | 0.17 |
| Mortality, n (%) | 155 (3.5) | 62 (4.4) | 93 (3.1) | 0.035 |
| Advanced respiratory support or mortality, n (%) | 437 (14) | 94 (11) | 343 (16) | <0.001 |

Sample size is n=3014 for advanced respiratory support or mortality outcome.

**Supplementary table 3: Sensitivity Individualized Treatment rule model results – excluding patients with COPD**

|  | Observed practice | Hypothetical: no steroids | Hypothetical: all treated with steroids | Optimal treatment | CRP regimen^a^ | CRP and glucose regimen^b^ | Advanced respiratory support or mortality model^c^ |
| --- | --- | --- | --- | --- | --- | --- | --- |
| Hospital-free days | 21.51 (21.26, 21.75) | 21.45 (21.21, 21.69) | 21.82 (21.57, 22.07) | 22.46 (22.23, 22.70) | 21.65 (21.40, 21.90) | 21.73 (21.49, 21.97) | 21.87 (21.63, 22.12) |
| Ventilation-free days | 26.62 (26.44, 26.80) | 26.73 (26.57, 26.89) | 26.64 (26.42, 26.86) | 27.50 (27.32, 27.67) | 26.74 (26.56, 26.92) | 26.78 (26.60, 26.95) | 26.70 (26.50, 26.90) |
| ICU-free days | 26.54 (26.38, 26.71) | 26.59 (26.44, 26.73) | 26.38 (26.18, 26.57) | 27.19 (27.04, 27.35) | 26.58 (26.42, 26.75) | 26.62 (26.45 (26.79) | 26.58 (26.40, 26.76) |
| Oxygen-free days | 24.57 (24.35, 24.79) | 24.58 (24.36, 24.80) | 24.81 (24.57, 25.05) | 25.60 (25.38, 25.81) | 24.73 (24.50, 24.95) | 24.79 (24.57, 25.02) | 24.89 (24.66, 25.12) |
| Mortality (%) | 3.79 (3.16, 4.42) | 3.35 (2.83, 3.87) | 4.06 (3.32, 4.80) | 1.70 (1.36, 2.03) | 3.34 (2.65, 4.03) | 3.30 (2.68, 3.91) | 3.96 (3.26, 4.67) |
| Advanced respiratory support or mortality (%) | 13.87 (12.42, 15.33) | 15.12 (13.65, 16.58) | 10.95 (9.20, 12.71) | 7.96 (6.74, 9.18) | 14.37 (12.81, 15.94) | 13.84 (12.24, 15.45) | 9.23 (7.87, 10.58) |

Estimates summarized using mean [Confidence interval (CI)] for continuous variables and frequency (percentage) for categorical variables. ^a^ Final model 1 based on CRP only by ITR regimen published by Smit et al (21). ^b^ Final model 3 based on CRP and glucose by ITR regimen published by Smit et al (21). ^c^ Final model advanced respiratory support or mortality based on entire study sample (not excluding patients with COPD).

**Supplementary table 4: Sensitivity Individualized Treatment rule model results – excluding patients with COPD and late steroids**

|  | Observed practice | Hypothetical: no steroids | Hypothetical: all treated with steroids | Optimal treatment | CRP regimen^a^ | CRP and glucose regimen^b^ | Advanced respiratory support or mortality model ^c^ |
| --- | --- | --- | --- | --- | --- | --- | --- |
| Hospital-free days | 22.21 (21.99, 22.42) | 22.17 (21.96, 22.39) | 23.17 (22.93, 23.41) | 23.47 (23.26, 23.68) | 22.39 (22.17, 22.61) | 22.53 (22.29, 22.76) | 22.70 (22.47, 22.94) |
| Ventilation-free days | 27.12 (26.97, 27.26) | 27.09 (26.95, 27.24) | 27.91 (27.80, 28.00) | 27.91 (27.80, 28.00) | 27.27 (27.11, 27.43) | 27.37 (27.22, 27.52) | 27.62 (27.50, 27.75) |
| ICU-free days | 26.95 (26.82, 27.08) | 26.93 (26.80, 27.06) | 27.67 (27.58, 27.76) | 27.67 (27.58, 27.76) | 27.09 (26.95, 27.23) | 27.18 (27.05, 27.31) | 27.41 (27.30, 27.52) |
| Oxygen-free days | 25.27 (25.09, 25.46) | 25.23 (25.04, 25.42) | 27.12 (26.95, 27.28) | 27.12 (26.95, 27.28) | 25.58 (25.35, 25.81) | 25.82 (25.60, 26.04) | 26.54 (26.36, 26.72) |
| Mortality (%) | 2.90 (2.33, 3.47) | 3.06 (2.46, 3.66) | 0.84 (0.59, 1.12) | 0.84 (0.59, 1.12) | 2.21 (1.53, 2.88) | 2.05 (1.45, 2.65) | 1.41 (0.97, 1.84) |
| Advanced respiratory support or mortality (%) | 12.94 (11.55 (14.33) | 12.83 (11.47, 14.20) | 10.85 (9.23, 12.47) | 2.51 (1.88, 3.14) | 12.19 (10.63, 13.74) | 11.62 (10.12, 13.12) | 10.94 (9.36, 12.51) |

Estimates summarized using mean [Confidence interval (CI)] for continuous variables and frequency (percentage) for categorical variables. ^a^ Final model 1 based on CRP only by ITR regimen published by Smit et al (21). ^b^ Final model 3 based on CRP and glucose by ITR regimen published by Smit et al (21). ^c^ Final model advanced respiratory support or mortality based on entire study sample (not excluding patients with COPD).

Supplementary table 5. Estimated ITR Model for Hospital-Free Days.

| Variable | Coefficient |
| --- | --- |
| Age at admit | -0.0182 |
| Weight | 0.0054 |
| WBC | 0.0248 |
| Eosinophils | -0.5617 |
| BUN | -0.0057 |
| PSI score | -0.0091 |
| Asthma | 0.1606 |
| CHF | -0.0881 |
| COPD | 0.6980 |
| ICU admit at 6hrs | 0.9476 |
| Neoplastic disease | 0.2147 |
| PaO2 | 0.0079 |
| SOFA score | 0.1436 |
| Altered mental status | 0.6612 |
| Gender (male) | 0.4591 |
| Bicarb | 0.0465 |
| Vasopressors within 6hrs of admission | -2.6037 |
| Liver disease | -1.4954 |
| NL ratio | -0.0282 |

The estimated treatment rule can be applied to a particular patient by multiplying covariates by the coefficient and taking the sum as a linear predictor (LP = Age at admit*(-0.0182) + Weight*(0.0054) + WBC*(0.0248) + Eosinophils*(-0.5617) + BUN*(-0.0057) + PSI score*(-0.0091) + Asthma*(0.1606) + CHF*(-0.0881) + COPD*(0.6980) + ICU admit at 6hrs*(0.9476) + Neoplastic disease*(0.2147) + PaO2*(0.0079) + SOFA score*(0.1436) + Altered mental status*(0.6612) + Gender (male)*(0.4591) + Bicarb*(0.0465) + Vasopressors within 6hrs of admission*(-2.6037) + Liver disease*(-1.4954) + NL ratio*(-0.0282)). If LP>0, the model recommends early steroids, if LP<0 the model recommends no early steroids.

Supplementary figure 1


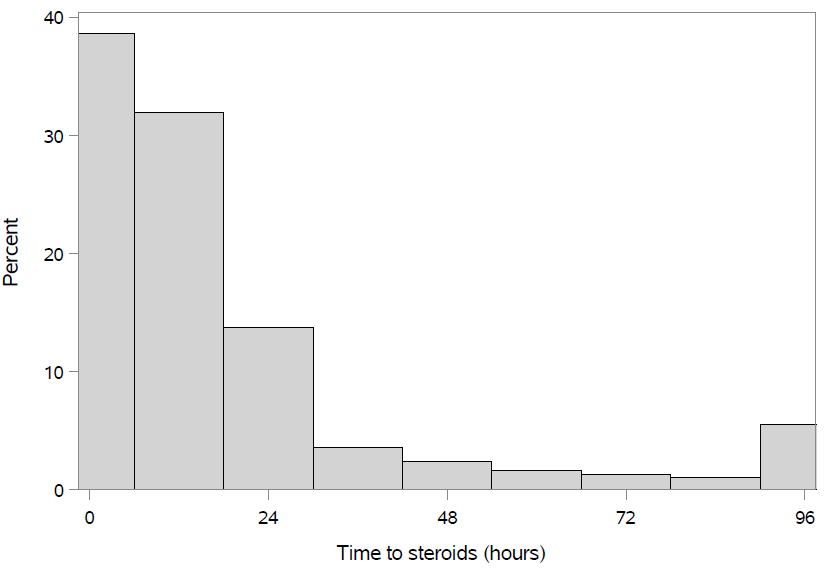

Supplement: Supplementary file 2 — Supplementary Material 2 [file 41479_2025_182_MOESM2_ESM.docx]
